# Research progress of porcine epidemic diarrhea virus S protein

**DOI:** 10.3389/fmicb.2024.1396894

**Published:** 2024-05-30

**Authors:** Haojian Luo, Zhaoping Liang, Junjie Lin, Yiqiao Wang, Yingying Liu, Kun Mei, Mengmeng Zhao, Shujian Huang

**Affiliations:** ^1^School of Life Science and Engineering, Foshan University, Foshan, China; ^2^College of Veterinary Medicine, South China Agricultural University, Guangzhou, China; ^3^Guangdong Hua Sheng Biotechnology Co., Ltd, Guangzhou, China

**Keywords:** porcine epidemic diarrhea, S protein, trypsin, porcine aminopeptidase N, genetic variation

## Abstract

Porcine epidemic diarrhea virus (PEDV) is a single-stranded RNA virus with a capsid membrane that causes acute infectious gastrointestinal disease characterized by vomiting, diarrhea, and dehydration in swine. Piglets are more susceptible to PEDV than adults, with an infection rate reaching 90% and a fatality rate as high as 100%. Moreover, PEDV has a rapid transmission rate and broad transmission range. Consequently, PEDV has caused considerable economic losses and negatively impacted the sustainability of the pig industry. The surface spike (S) glycoprotein is the largest structural protein in PEDV virions and is closely associated with host cell fusion and virus invasion. As such, the S protein is an important target for vaccine development. In this article, we review the genetic variation, immunity, apoptosis-induction function, virulence, vaccine potential, and other aspects of the PEDV S protein. This review provides a theoretical foundation for preventing and controlling PEDV infection and serves as a valuable resource for further research and development of PEDV vaccines.

## 1 Introduction

Porcine epidemic diarrhea (PED) is a swine illness caused by the porcine epidemic diarrhea virus (PEDV). Common clinical manifestations include emesis, diarrhea, desiccation, and anorexia. Newborn piglets (up to 10 days) are particularly susceptible to acute and highly infectious intestinal diseases upon PEDV infection, with a mortality rate that can reach 100%. Therefore, PED is also known as epidemic diarrhea in piglets, which contributes to notable economic losses for the swine industry.

PEDV was first identified in China in the 1980s and a subsequent outbreak occurred in the United States in 2013. Until 1984, PEDV was identified in confirmed cases of PED using fluorescence-labeled antibodies and serum neutralization tests (Belouzard et al., 2012). In 2010, mutant PEDV strains emerged that were responsible for causing large-scale outbreaks. A recent analysis of clinical diarrhea samples from pigs concluded that PEDV has the highest positive rate in samples from piglets, with a detection rate that markedly exceeds that of other viruses, including porcine rotavirus and porcine delta-coronavirus (Zhang, 2023). These results implicate PEDV as the primary viral pathogen causing diarrhea in piglets. Accordingly, efforts have been dedicated toward improving the prevention and control of PEDV to enhance the growth performance of piglets.

In 1988, Hofmann and Wyler (1988) and Lawrence et al. (2014), a research group from Sweden, were the first to successfully culture PEDV in African green monkey kidney cell lines (Vero and MARC-145) by supplementing the growth medium with trypsin. The cells underwent pathological changes after five consecutive generations. Subsequently, researchers in various countries have successfully cultured PEDV using primary cells from the bladder and kidney of piglets and have passaged cells from the IB-RS-2, KSEK6, ESK, CPK, and HEK-293 T cell lines (Kadoi et al., 2002).

PEDV is an alpha-coronavirus in the Nidoviridae family within the subfamilies Coronavirus and Alpha-Coronavirus. PEDV is a positive-sense RNA virus with spherical particles measuring approximately 95–190 nm in diameter, comprising genomic RNA and a nuclear capsid enclosed in a capsule membrane on which radially arranged 18–23 nm spikes are present. The complete PEDV genome is approximately 28 kb (Han, 2014) and encodes 17 non-structural proteins (NSP1–16 and ORF3) and four structural proteins: the spike (S), envelope (E), membrane (M), and nucleocapsid (N) proteins (Kocherhans et al., 2001). PEDV and other pathogenic coronaviruses have been detected in domestic animals and humans, with the capacity to cross the host species barrier (Li et al., 2016).

The S protein of PEDV is a type-I transmembrane glycoprotein located on the outer surface of the viral particles comprising 1,383 amino acids (aa), with an estimated molecular weight of 150–220 kDa (Park et al., 2007). Electron microscopy has revealed that the approximately 20 nm S protein exhibits a rod-like radiocoronal morphology on the virion surface. The S protein is cleaved at potential N-glycosylation sites into the S1 (aa 1–789) and S2 (aa 790–1,383) subunits by foreign or host cell proteases (Lee et al., 2010). S1 comprises the N-terminal domain (S1-NTD) and the C-terminal domain (S1-CTD) (Li, 2012), essential for recognizing and binding host cell receptors. Meanwhile, the S2 region can be divided into three subregions: the extracellular region, transmembrane anchor region, and cytoplasmic tail (Sun, 2019). The cytoplasmic tail contains the primary neutralizing epitopes of the virus and plays a crucial role in mediating the fusion of the virus with the host cell membrane (Brian and Baric, 2005). The first 18 aa of the S1 subunit serve as signal peptides and are located in the NTD; the subunit contains an additional 790 aa; the S2 subunit comprises 1,383 aa, comprising transmembrane regions. The S protein exists in the form of trimers in PEDV particles (Song and Park, 2012). These are determined by the presence of multiple epitopes that play crucial roles in eliciting neutralizing antibodies in a living organism. However, the high variability of the NTD of the S1 subunit presents a remarkable challenge for vaccine development, complicating efforts to prevent and control PEDV (Chen et al., 2016).

The primary mode of PED transmission is the fecal–oral route. The virus enters the pig intestine through the mouth and multiplies within the intestinal epithelial cells (Sun et al., 2012). However, PEDV has also been detected in sow milk and alveolar macrophages, suggesting the possibility of vertical and respiratory transmission modes (Park and Shin, 2014). PEDV can also be transmitted via aerosol particles (Lee, 2016). Indeed, PEDV reportedly has the potential to cause diarrhea through nasal transmission. In this scenario, PEDV enters the nasal environment, where it contacts epithelial cells. Infected subcutaneous dendritic cells (DCs) transmit PEDV to peripheral blood helper lymphocytes through viral synapses. Finally, the virus contacts intestinal epithelial cells via blood circulation, leading to intestinal infection in piglets (Li et al., 2018).

PEDV primarily replicates in small intestine villi and causes pathological alterations in epithelial cells. Vacuolation of the intestinal epithelial cells leads to apoptosis, resulting in the release of decompositional enzymes into the lumen. Consequently, a notable decrease in enzyme secretion, thinning of the intestinal wall, and a decrease in the surface area available for absorption occur, which weakens the absorption of intestinal nutrients, leading to food fermentation and irregular intestinal peristalsis (Wang X. K., 2014; Zhang Y., 2022). Since PEDV infection in piglets prior to weaning causes a decrease in digestive enzyme activity, intestinal lactose accumulates along with changes in inner and outer osmotic pressure, ultimately causing osmotic diarrhea (Jung et al., 2006). A study of the intestinal pathology of pigs infected with PEDV detected inflammatory lesions in the mesenteric lymph nodes; the mucosal layer exhibited inflammatory infiltration of lymphocytes, neutrophils, and other cells; and the intestinal villi underwent atrophy with sparse microvilli that shed irregularly (Jung et al., 2006). Vacuoles also begin to appear in dense intestinal epithelial cells, leading to morphological changes. Nuclear shrinkage occurs at the tip of the intestinal villi and most PEDV lesions occur in the jejunum and ileum (Sueyoshi et al., 1995). Specifically, in cases of acute infection in nursery pigs, virions are primarily observed in the middle jejunum and ileum, followed by the proximal jejunum, distal jejunum, and duodenum (Jung et al., 2018). However, PEDV antigens are commonly observed in the intestinal crypt cells, lamina propria, Peyer’s plaques, and antigen-presenting cells (APCs), such as macrophages. These APCs may facilitate viral penetration of the intestinal epithelial barrier, resulting in viremia (Li et al., 2019). When nursing pigs are acutely infected with PEDV, the virus is initially detectable at the junction between the villi and crypts in the small intestine and subsequently spread to the upper portion of the jejunum. Within 24 h of oral inoculation, the entire villous epithelium of the jejunum and ileum becomes progressively affected. The vessels at the villus–recess interface near the submucosa have been hypothesized to serve as the route for PEDV to spread from the acutely infected intestine to the bloodstream (Jung et al., 2018; Zhang X. Q., 2022).

In summary, PED is a disease with rapid transmission that is responsible for causing considerable harm to the pig industry. Meanwhile, the surface S protein has been identified as a crucial target for designing preventative and treatment strategies. This article reviews the current status of the PEDV S protein from multiple aspects and proposes prospects for future research directions in the context of the S protein.

## 2 S protein genetic variation

Based on phylogenetic analysis of the complete S gene sequence, PEDV can be classified into two distinct genotypes, genotype 1(G1) and genotype 2(G2). Lee (2015) suggested that each large group should be further divided into two subgroups. That is, G1 is categorized into two subgroups: G1a and G1b. This includes the classic strain and its vaccine strain, as well as the SINDEL strain named by American researchers. Similarly, G2 strains are divided into G2a and G2b subtypes; the latter may be caused by a point mutation in the G2a subtype. Although no clear distinction has been established between the two strains, they could be distinguished based on their geographical site of isolation. The G2a subtype primarily includes strains that emerged during early outbreaks in Asia, whereas the G2b subtype predominantly comprises strains currently circulating in the US and Asia (Lee, 2015).

Sun et al. (2015) analyzed genetic variation in 49 PEDV strains with sequences in the GenBank database and identified four highly variable regions in the PEDV genome. Using AH2012 (GenBank: KC210145) as the reference sequence, the start and end positions of the V1 region were located at 1721–3500 bp. The V2 region spans the 20,661–22,300 bp position and carries the PEDV S1 gene. The V3 zone spans the 23,541–25,200 bp region and carries the S2 gene, while the V4 zone spans the 26,741–27,700 bp region (Sun et al., 2015). Most variation in the PEDV genome is distributed within the S gene, representing a substantial contribution to the overall genetic diversity of PEDV.

In particular, notable variations are present in the S gene between the G1 and G2 strains. According to a multi-sequence comparison analysis of the PEDV S protein, among the hundreds of aa differences between the G1 and G2 strains, most occur within the S1 gene. Conversely, the S2 gene shows a relatively high degree of conservation (Deng, 2017). In addition to base substitutions, base insertions, and deletions also occur between the S genes of the G1 and G2 strains. Recently, strains with large fragments missing at other aa sites of the S protein have been isolated from wild-type viruses. For example, the Korean strain MF3809/2008 (GenBank: KF779469) carries a 204 aa deletion in the S protein (Park et al., 2014). Meanwhile, the Japanese strain Tottori2/JPN/2014 (GenBank: LC022792) has a continuous 194 aa deletion at the N-terminus of the S protein that weakens the virulence and indicates a close genetic relationship with the G2 strain (Murakami et al., 2015). Multiple S1 NTD-deletion strains co-infected with the US prototype were also identified in outbreaks occurring in 2016 and 2017 (Su et al., 2018).

Since the outbreak of mutated PEDV strains in 2010, the variability of the S gene has progressively increased, suggesting a high degree of diversity within the PEDV genome. In addition, the S protein encoded by this gene contains four important neutralizing antigen epitopes. Therefore, studying the S gene has notable implications for understanding the genetic diversity of PEDV. A common G2 PEDV variant is considered to have originated from South Korea; however, few studies assessed the origin and base replacement rate of the common ancestor strain. Ao (2020) collected 898 samples from 18 provinces and cities in China between 2016 and 2018 to understand the present epidemiological characteristics of PED and predict the future genetic variation trends of PEDV. They selected PEDV strains with notably positive characteristics from various geographical regions and historical periods for comprehensive sequencing of the S gene (Ao, 2020). Initially, the samples were compared with the S gene sequence of reference strains in GenBank, demonstrating that the sequence consistency of the sample strain was higher than that of the reference G2 strains YN144, FL2013, CH/HNQX-3/14, and AJ1102. In addition, sequence consistency with the G1 strains CV777, LZC, attenuated CV777, and ZL29 was low. They further used 17 other full-length sequences of the PEDV S gene isolated from different countries as reference strains and 45 full-length sequences of the PEDV S gene were used to construct a neighbor-joining phylogenetic tree.

Their study revealed that among the 45 PEDV strains, 44 belonged to the G2-a subtype, whereas one (V7-HB2018) belonged to the G1-b subtype (Ao, 2020). Furthermore, the 44 G2-a subtype strains were categorized into three clusters based on an S gene neighbor-joining phylogenetic tree: G2-a1, G2-a2, and G2-a3. The 17 sequenced strains, including E3-SX2017, V1-HB2018, and E6-SN2017, belonged to the G2-a1 cluster and these clusters were closely related to strains XM2-4 and HBXY1, with sequence similarities ranging from 95.7 to 99.5%. Strains Y1-HA2018, B2-HB2017, B5-HB2017, and Z11-HA2018 were in the G2-a2 cluster, with close relationships to strains CH-SCZG-2015 and JS201603. A total of 23 strains, including M3-SX2017, G2-HE2017, and Z7-HA2018, belonged to the G2-a3 cluster, which was more closely related to strains AJ1102 and YN1, with a sequence similarity ranging from 95.8 to 99.6%. These findings indicated that the mutant strain remains the primary strain associated with PED in pigs across various countries. This was further supported by separate studies conducted by Grasland et al. (2015) and Guo et al. (2022). Additionally, Xiong et al. (2023) collected 340 specimens suspected of being contaminated with PEDV between 2020 and 2021. PEDV antigen analysis and sequencing were conducted and the results were compared to reference sequences in GenBank. They found that G1 and G2 PEDV strains were prevalent in some areas of China from 2020 to 2021, with G2 strains being predominant; however, the prevalence and variation patterns of PEDV are complex. Shi et al. (2023) sequenced 525 clinical samples collected from 2020 to 2021, revealing that G2 was the predominant epidemic genotype of PEDV.

Ao (2020) also utilized DataMonkey to analyze positive selection sites in each structural gene of the G2 PEDV strain. They discovered three positive selection sites on the S protein, specifically in the overlapping regions of S1–NTD, S1–CTD, and S1–CTD. Adaptive mutations at these three positively selected sites may enhance viral infectivity, help the virus evade host immunity, and enable continual environmental adaptation (Ao, 2020). A study conducted in 2022 on the evolution of the molecular structure of PEDV confirmed the presence of these three positively selected nucleic acid sites and their impact on virus virulence (Huang et al., 2022).

Cheng et al. (2023) amplified the S1 gene sequence of 11 PEDV endemic strains isolated from Zhejiang from 2015 to 2017 by RT-PCR and compared the amino acid sequence variations of the S1 protein. They reported that 92% of the 97 PEDV strains were G2, of which 33% of the prevalent strains belonged to G2a and 67% were G2b, of which 15% belonged to the G2a subtype and 85% belonged to the G2b subtype. Meanwhile, the epidemic and vaccine strains in Shandong from 2017 to 2018, such as CV777, belonged to the G1 type. Compared with the classic CV777 strain, mutations were detected at eight amino acid sites (A517S, S523G, V527I, T549S, G594S, A605E/D, L612F/Y, and I635V) with the CO-26 K equivalent epitope COE. However, although no amino acid variations were detected in the SS2 region, the SS6 region carried one amino acid mutation (Y766S) and novel mutation sites (T636I and S749G) appeared after 2018. Furthermore, Zhao et al. (2023) collected disease samples from 16 provinces and cities in China and analyzed the amino acid sequences of the S protein of 76 strains. Compared with the vaccine strain CV777, the S gene sequence of 71 GIIB strains had highly similar genetic markers to the GII-type representative strains, including four amino acid insertions between 58 and 59 amino acids. One amino acid was inserted between positions 139 and 140; the S gene sequence of five strains had the same genetic marker as the representative S-INDEL type strain; one amino acid deletion was detected between positions 139 and 140; and several continuous mutations were identified in the N terminal of S protein. In addition, compared with other genotypes, ZJLS10-2021 and GXNN14-2021 strains had one amino acid insertion between 1,267 and 1,268 amino acids and three amino acid insertions between 379 and 383 amino acids, respectively. Hence, the PEDV S gene was readily mutated in different provinces and cities in China, and the major circulating strains in the same region have relative genetic stability.

## 3 Interaction between S protein and host protein

PEDV shows a specific preference for replication in the villous epithelial cells and intestinal cells of the porcine small intestine, indicating a unique tissue tropism. Porcine aminopeptidase N (pAPN), which is mainly expressed in the epithelial cells of the small intestine, serves as a receptor for PEDV. The N-terminal region of the functional domain of the PEDV S1 protein is crucial for pAPN receptor recognition (Deng et al., 2016). The first step of PEDV invasion involves the S protein attracting pAPN to the host cell surface; pAPN clusters around the S protein and facilitates membrane fusion between the viral envelope and host cell membrane. Consequently, the virus enters the host cell by releasing its genome into the cytoplasm. The viral genome is then promptly translated into replicases 1a and 1ab, polymeric proteins that can be enzymatically cleaved into 16 non-structural proteins containing a replication and transcription complex. The viral genome is then used to synthesize negative-sense RNA (Cong et al., 2015). The N protein interacts with the newly formed genomic RNA to create a helical ribonucleoprotein complex assembled through budding and released via exocytosis (Li et al., 2009; Lee, 2015).

**Figure 1:**
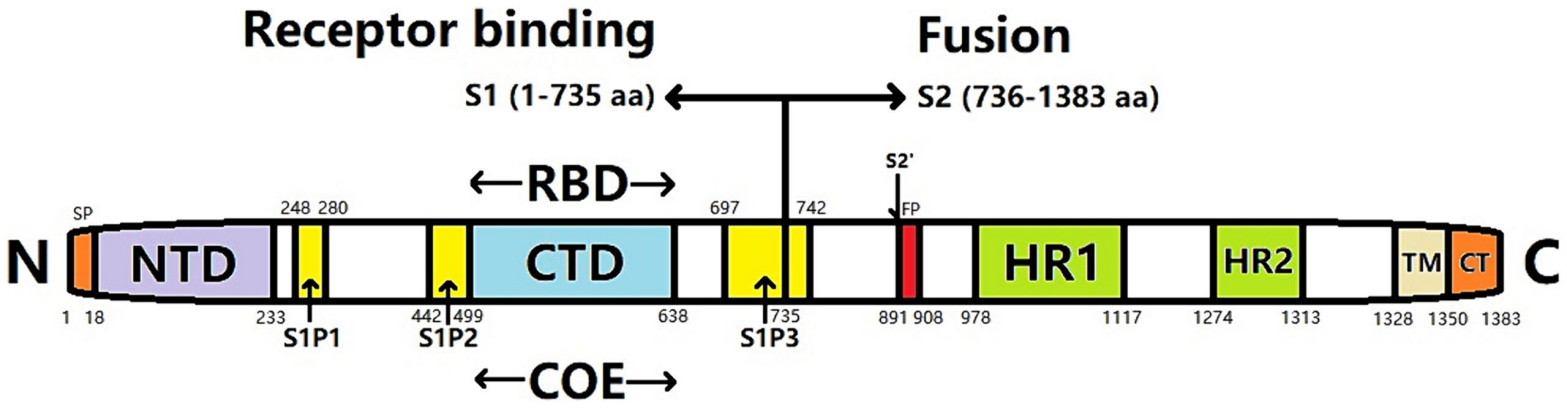
Gene structure of PEDV spike (S) protein. SP, signal peptide; NTD, N-terminal domain; CTD, C-terminal domain; RBD, receptor binding domain; COE, CO-26 K equivalent; FP, Fusion peptide; HR1, heptapeptide-repeat region 1; HR2, heptapeptide-repeat region 2; TM, transmembrane domain; CT, cytoplasmic tail; S1P1, S1P2, S1P3, S1 subunit linear antigen epitope.

The S protein of PEDV comprises two subunits: the N-terminus of the S1 subunit and the C-terminus of the S2 subunit. The S1 subunit contains a receptor-binding domain (RBD) that is responsible for receptor binding, whereas the S2 subunit contains a hydrophobic fusion peptide and two heptapeptide-repeating regions (HR1 and HR2) (Figure 1). The S2 subunit has a coiled helical structure that mediates membrane fusion between viruses and cells (Li et al., 2016). S-protein monomers can self-assemble into a trimeric spatial conformation and anchor to the viral envelope. Attachment of the RBD to the cellular receptor triggers structural changes in the S1 and S2 subunits that cause the exposed fused loop to be incorporated into the membrane of the target cell. Subsequently, the HR1 and HR2 regions of the S-glycoprotein trimer create a six-helical bundle configuration that tightly connects the membranes of the viral and host cells, facilitating their fusion (Xie, 2020).

Several receptors facilitate the invasion of host cells by PEDV virions through contact with the S protein, including pAPN, and sugar is a PEDV co-receptor (Liu et al., 2015; Narayanan et al., 2015). Oh et al. (2003) showed that PEDV can selectively attach to pAPN; however, this can be hindered by anti-PAPN antibodies, indicating that pAPN functions as a cellular receptor for PEDV (Oh et al., 2003). Li et al. (2007) transferred the pAPN gene into Madin–Darby canine kidney (MDCK) cells insensitive to PEDV. PEDV was observed to replicate rapidly and continuously in MDCK cells that expressed pAPN, whereas infection was blocked by treatment with an anti-pAPN antibody. These findings confirmed that pAPN serves as a cellular receptor for PEDV (Li et al., 2007).

By studying swine testicular cells (ST cells) that stably express pAPN receptors, Nam and Lee (2010) demonstrated that receptor density is crucial to efficiently infecting ST cells with PEDV. This also accounts for why PEDV cannot replicate in normal ST cells that express pAPN receptors. Other studies have confirmed that transgenic pAPN mice can be infected with PEDV (Park et al., 2015). Liu et al. (2015) revealed that PEDV can recognize porcine and human APN as a receptor and utilizes sugar as a co-receptor. Subsequent research indicated that the C-terminal region of the PEDV S1 protein (499–638 aa) can interact with pAPN (Deng et al., 2016).

However, there has been debate regarding whether pAPN is a genuine receptor for PEDV. Recent research suggests that pAPN does not function as a cellular receptor for PEDV, although its activity may facilitate viral infection (Shirato et al., 2016). Disruption of APN in porcine ST cells and various human cell lines, such as liver cancer (Huh7 cells), cervical cancer, and HeLa cells, using CRISPR/Cas9 genome-editing technology does not affect the susceptibility of cells to PEDV (Li et al., 2017). Similar results have been reported for Vero cells (Ji et al., 2018), frequently used to isolate PEDV. However, Vero cells do not express pAPN. As a result, the question of whether pAPN serves as a PEDV receptor has sparked notable debate in this research field (Li et al., 2020).

To clarify the interaction properties between pAPN and the PEDV S protein, Xie (2020) examined the interplay between S protein and pAPN through various experimental methods, including liposomal transfection, western blotting, pull-down assays, immunofluorescence assays, and others. The region responsible for the interaction between the S and pAPN proteins of the PEDV epidemic strain SHpd/2012 was found to be located within the 249–529 aa region in the functional domain. Furthermore, positions 249 aa and 529 aa were key sites for this interaction. These findings indicate that pAPN is capable of interacting with the S protein. However, the fact that Vero cells do not express the pAPN protein (Kusanagi et al., 1992) yet facilitate the successful proliferation of PEDV contradicts the assertion that pAPN serves as a cellular receptor for PEDV. Therefore, the invasion mechanism and pathways of PEDV can be inferred to vary in different cell types. Kamau et al. (2017) confirmed that pAPN domain VII is significantly involved in the binding of PEDV to receptors and its invasion.

In addition, trypsin can enhance the proliferation of PEDV in Vero cells by facilitating virus invasion and release. During the initial stage of viral entry, trypsin increases the permeability of PEDV bound to Vero cells by approximately two-fold. However, trypsin treatment of the virus does not enhance its infectivity before it binds to its receptor. Hence, binding of the virus to its receptor is necessary for trypsin-mediated entry of the virus after PEDV infection (Park et al., 2011). Trypsin induces significant cytopathic changes in infected cells during the budding phase of viral infection, including cleavage of the S protein in receptor-bound virions though not in free virions. This cleavage of the PEDV S protein increases susceptibility and syncytia formation in Vero cells infected with PEDV (Shi, 2018).

In summary, although pAPN may not function as a receptor for PEDV, it enhances viral replication and proliferation through a synergistic effect with an as-yet-unidentified receptor (Shirato et al., 2016). Moreover, the presence of trypsin enhances the synergistic effect between pAPN and its receptors, promoting the replication and proliferation of PEDV (Oh et al., 2019). Trypsin is a serine endopeptidase that has been widely studied with respect to the cleavage and activation mechanisms of viral glycoproteins. The optimal pH for pancreatic enzymes is approximately 8.0 and they exhibit high lytic activity toward arginine (R) or lysine (K) residues. Most viruses easily undergo lysis at arginine sites. However, only a limited number of pancreatic cleavage sites are exposed in the S proteins of coronaviruses. Pancreatic enzymes are commonly present in the respiratory tract; however, their capacity to break down substances is often impeded by alpha-1 anti-trypsin. Most of the coronavirus S proteins in the intestine are cleaved and activated by trypsin, which is secreted by the epithelium of the small intestine (Millet and Whittaker, 2015). This action can also improve the functionality of the S protein and facilitate membrane fusion. Additionally, most PEDV strains prefer pancreatic enzymes. Meanwhile, pancreatic enzymes in Vero cells play a lytic role only after the virus binds to the receptor, not in the virion itself (Park et al., 2011). Therefore, the promoting effect of pAPN on PEDV results from the interaction between pAPN, S protein, and trypsin. However, the specific mechanism of action of pAPN in PEDV infections remains unclear. Elucidating this mechanism and identifying the PEDV receptor are the primary research objectives for achieving stable isolation of the virus.

Li (2022) successfully purified the PEDV–S and PEDV–S1 fusion proteins. The α-subunits of sodium/potassium transport ATPase, GRP75, GRP78, and GRP60 were obtained by proteomic analysis. Moreover, the 60 kDa chaperone protein, cell division control protein 42 homolog (CDC42), ATP synthase α-subunit, ATP synthase β-subunit, prohibitin, and Annexin9 are Vero cell membrane proteins that form interactions with the PEDV–S1 protein, suggesting candidates of potential receptors in PEDV-infected cells (Li, 2022).

To date, most international research in the field of PEDV has focused primarily on production practices and applications to prevent disease transmission and spread. Meanwhile, experimental research has focused on diagnosing PEDV and developing vaccines. However, current research on the cellular infection mechanism of viruses and the interplay between the viral and receptor proteins is at an early stage. In particular, literature is scarce regarding the interaction between the S protein and other proteins specific to PEDV. Consequently, investigating the interaction mechanism between the S protein and other PEDV proteins while examining the interaction between the S protein and host receptor proteins such as pAPN, is imperative.

## 4 PEDV S protein induces cell apoptosis

Viral infections are associated with the induction or inhibition of host cellular apoptosis. Induction of apoptosis is primarily a protective mechanism in cells; hence, by actively inhibiting apoptosis, the virus facilitates its own replication and proliferation in host cells (Esolen et al., 1995). Apoptosis, mediated by the immune system, maintains the normal functioning of the body and helps to continuously eliminate viruses. However, apoptosis is a double-edged sword phenomenon; although it can eliminate the virus in the host, it can also be exploited by the virus to induce cytopathic effects that facilitate viral release (Duan et al., 2021; Sixt, 2021). In the intestinal cells, an elevated rate of apoptosis may undermine the structural integrity of the intestinal barrier (Chen and Macara, 2005). This is particularly relevant for PEDV, which primarily infects the intestinal tract of pigs.

Numerous viruses can actively trigger cellular apoptosis, facilitating the dissemination and transmission of progeny viruses to adjacent cells (Neumann et al., 2015). The pro-apoptotic effect significantly contributes to cell and tissue damage, enhancing disease severity in piglets infected with PEDV. Kim and Lee (2014) used the terminal deoxynucleotidyl transferase dUTP nick-end labeling assay to detect apoptotic markers in the duodenum, jejunum, and ileum of PEDV-infected pigs. The results demonstrated that PEDV infection induces apoptosis. In addition, *in vitro* infection of Vero cells with PEDV induces apoptosis. However, the addition of caspase inhibitors did not affect the PEDV-induced apoptosis in Vero cells. Further analysis of the mitochondrial apoptosis-inducing factor (AIF) using immunofluorescence and western blotting revealed that PEDV-infected cells significantly enhance AIF transfer. Conversely, treatment with AIF inhibitors significantly inhibits PEDV-induced apoptosis (Kim and Lee, 2014). Overall, this evidence indicates that PEDV induces apoptosis through an AIF-mediated pathway independent of caspases and this mechanism plays an important role in facilitating PEDV replication and the development of pathogenic effects (Chen et al., 2018).

The S protein, located on the surface of PEDV, serves as a binding receptor and plays a crucial role in the initiation of apoptosis induced by PEDV. These effects constitute the primary focus of current research endeavors on PEDV. Tan et al. (2021) confirmed this mechanism by comparing the S proteins of two PEDV strains, YN200 and DR13 and reviewing related studies on PEDV and Vero-E6 cells. They found that the S protein region of PEDV induces apoptosis in Vero-E6 cells and that the S1 subunit may significantly impact the induction of apoptosis by PEDV (Tan et al., 2021). Chen et al. (2018) constructed protein expression plasmids for each PEDV protein and used western blotting to study their effects on apoptosis; they found that PEDV infection results in notable cytopathy and apoptosis in Vero-E6 and Marc-145 host cells. This confirmed the dominant role of the S1 subunit as a key functional protein in promoting apoptosis (Chen et al., 2018). Therefore, the S1 protein has the potential to trigger apoptosis in response to the coronavirus, suggesting that inhibition of PEDV infection and the promotion of apoptosis by targeting S1 protein expression could be a viable approach for preventing and managing PEDV.

## 5 Influence of S protein on PEDV virulence

The V672F substitution of PEDV S protein increases Vero cell fusion and viral replication (Wanitchang et al., 2019). This mutation is also associated with viral adaptation to growth conditions in a laboratory setting and a reduced ability to cause disease in living organisms (Sato et al., 2011). This suggests that the PEDV S gene plays an important role as a virulence factor (Wang et al., 2018). Mutations in the S protein can cause changes in the *in vitro* culture ability and virulence of PEDV. Therefore, S protein has been employed as a valuable element for examining genetic relationships between PEDV strains, identifying their epidemiological status, and for vaccine development (Fan, 2023).

Even single nucleotide mutations can alter the pathogenicity, immunogenicity, and tissue specificity of PEDV. The S protein functions as the primary envelope glycoprotein responsible for binding to receptors, along with roles in viral adherence, fusion with the cell membrane, and entry into host cells. Furthermore, the S protein can be divided into the S1 (1–789 aa) and S2 (790–1,383 aa) domains based on the similarity to the S proteins of other coronaviruses; variations in these regions are associated with adaptability to *in vitro* culture and reduced virulence *in vivo* (Sato et al., 2011). Recently, the global prevalence of PEDV infections have increased notably. These strains differ from previously reported highly virulent PEDV strains primarily based on insertions or deletions in the S gene (Oka et al., 2014). However, the S and ORF3 genes of PEDV show conspicuous genetic variability with varying levels of nucleotide insertions, deletions, and mutations among strains. These genetic variants are frequently used in epidemiological studies. Based on the genetic diversity and gene frequencies, PEDV can be categorized into two genotypes: G1 (classic) and G2 (endemic), which differ in their pathogenicity and virulence. Different PEDV genotypes can provide cross-protection. Nucleotide insertions or deletions in the S gene can also affect viral virulence (Goede et al., 2015).

During the interaction between PEDV and the host, localization of the PEDV S protein in the cell is primarily regulated by the YxxO motif at the C-terminus and endoplasmic reticulum cell signaling (ERCS). Loss of the YxxO motif and ERCS results in a notable increase in the quantity of S proteins exposed on the cellular surface, which facilitates cell membrane fusion and augments the recognition of immune cells. Additionally, this interaction interferes with the formation of S and M proteins in the endoplasmic reticulum–Golgi intermediate compartment. This ultimately results in the production of defective viruses with reduced virulence. For intact S proteins, the Yxxø motif and ERCS not only help S proteins to be absorbed into the cell from the cell surface to evade recognition by extracellular immune cells but further enable the virus to relocate to the endoplasmic reticulum and Golgi apparatus, where it undergoes assembly to generate completely developed virions (Hou et al., 2019).

Recent studies have compared the pathogenicity of PEDV strains without an insertion–deletion marker in the S protein (non-S INDEL PEDV) with that of strains containing an S gene insertion–deletion marker (S INDEL PEDV). Compared to the S protein sequence, partial mutation or deletion of its nucleotide sequence has been observed (Zhang et al., 2018). Non-S INDEL PEDV is generally referred to as highly virulent PEDV. Compared with S-INDEL PEDV, the infection and mortality rates are relatively high for non-S INDEL PEDV, with a fatality rate reaching 100% in seven-day-old piglets (Annamalai et al., 2017). Non-S INDEL PEDV has a fast transmission speed and rapid onset, causing more serious pathological changes, including intestinal villi atrophy and necrosis. Furthermore, there is a broad spectrum of ages at which infection can occur, with nearly all pigs susceptible to infection with non-S INDEL PEDV regardless of their age, although clinical symptoms are worse for pigs of younger ages. Adult pigs often experience temporary diarrhea, whereas suckling piglets exhibit diarrhea within the first day of a non-S INDEL PEDV infection and weaned pigs take longer to develop diarrhea symptoms (Jung et al., 2015). Compared to pigs infected with S-INDEL PEDV, weaned pigs exposed to non-S INDEL PEDV through oral or aerosol exposure have a significantly higher incidence of viremia (Gallien et al., 2018). In summary, the PEDV S protein is essential for determining the pathogenic nature of the virus. Suzuki et al. (2018) confirmed that the S1 subunit, which contains the D0 and S1 domains, is the major region of the S protein that determines viral virulence. In addition, the removal of the D0 domain can reduce viral pathogenicity (Suzuki et al., 2016). Furthermore, variants harboring the D0 deletion can penetrate host cells *in vivo* and *in vitro* (Oka et al., 2014).

The PEDV S protein can bind to specific receptors on host cells, promoting viral infection and enhancing virus virulence. Sun et al. (2009) used phage-display technology to screen the receptor-binding region of PEDV. The 249–529 aa within S1 were found to play a crucial role in the interaction of the virus with pAPN during the binding process (Sun et al., 2009). Subsequently, Deng et al. (2016) confirmed that the PEDV S1-CTD is the primary receptor recognition region of pAPN and that the S1-NTD recognizes co-receptor sugars. The classic weak PEDV strain CV777 and the mutant strain GHGD-01 exhibit minimal differences in pAPN binding ability. However, GHGD-01 exhibits stronger sugar recognition ability, suggesting that the sugar receptor protein on GHGD-01 has a higher binding affinity for the PEDV S protein. A mutation or deletion in the S gene of the PEDV-mutant strain augments its capacity to invade cells. This may also be a reason for the observed differences in virulence between strains (Deng et al., 2016). Subsequent research on the impact of PEDV S protein on virulence should focus on investigating the interaction between the S protein and the receptor proteins of the host cell. This line of inquiry could explore the binding mechanism and the capacity of the PEDV S protein to interact with host cell receptor proteins, improving our understanding of the impact of the S protein on PEDV virulence.

## 6 PEDV S protein mediates host immune responses and its application in vaccine research

The S protein of PEDV serves as the principal antigenic glycoprotein responsible for the viral invasion of host cells. S protein can identify and attach to receptors on the surface of host cells, with a pivotal role in the initial phase of virus invasion (Li et al., 2016). Pregnant sows vaccinated with the PEDV S1 protein can transfer passive immunity to unweaned piglets via the colostrum, helping them resist PEDV infection (Zhang X. Q., 2022). The S2 region at the C-terminal (736–1,383 aa) of the PEDV S protein is a transmembrane region that facilitates the entry of viral nucleic acids into host cells through membrane fusion (Li et al., 2007). Notably, a protease cleavage recognition site was identified at the S1 and S2 junction. The host protease can target this junction and the region upstream of the fusion peptide in the S2 region of S protein. This leads to a conformational change in the S protein, ultimately facilitating membrane fusion during the receptor-binding phase (Wicht et al., 2014).

With the continuous variation in PEDV strains, replacement, insertions, or deletions of the PEDV S1 protein (Vlasova et al., 2014; Yamamoto et al., 2015) have occurred. The PEDV S protein undergoes evolutionary changes to enhance its capacity to evade the host immune system while improving its receptor recognition and facilitating envelope fusion. Current vaccines are ineffective in providing adequate protection against PEDV epidemic strains (Islam et al., 2016). Therefore, to advance our understanding of the immune response mechanism of the PEDV S protein in host cells, further investigation of its immune reactivity in the S1-COE region and clarification of its epitope characteristics are necessary.

### 6.1 Multi-epitope vaccine

As the predominant structural protein located on the exterior of PEDV, the titer of S protein-specific antibodies is significantly correlated with the neutralizing activity of the antibodies produced by immunized or infected animals (Song et al., 2016). Therefore, determination of specific antigenic sites on the S protein, particularly neutralizing epitopes, is important for establishing PEDV detection methods and developing multi-epitope peptide vaccines. Zhang et al. (2021) employed an S-protein polyclonal antibody from an anti-PEDV CV777 vaccine strain to delineate the essential motifs for neutralizing linear B-cell epitopes within the intracellular domain of the S protein. They speculated that differences in adjacent or similar amino acid residues in this region of the circulating S protein caused the change or loss of immunogenicity. Furthermore, they found that the serum obtained from animals immunized against the S protein of the epidemic strain could not detect a short peptide containing the QPYE motif. This lack of recognition was attributed to the absence of corresponding antibody components (Zhang et al., 2021).

The S protein of PEDV includes a segment that aligns with the CO-26 K region within the transmissible gastroenteritis virus (TGEV) and is the COE that spans 499–638 aa. This region is the primary site responsible for neutralizing epitopes capable of stimulating the production of neutralizing antibodies (Chang et al., 2002). To date, a multitude of research efforts have been undertaken to identify the epitopes of neutralizing antibodies that target the S protein of PEDV. These studies revealed that distinct segments of the S protein, including 19–220 aa (Li et al., 2017), 499–638 aa (Chang et al., 2002), 636–789 aa (Sun et al., 2007), 744–759 aa, 747–774 aa, and/or 756–771 aa (Okda et al., 2017), 1,268–1,322 aa (Zhao et al., 2018), and 1,371–1,377 aa (Okda et al., 2017), can induce the host to generate antibodies with neutralizing properties. The 1368GPRLQPY1374 epitope, identified using phage-display technology, is located in the C-terminal region of the S protein and exhibits neutralizing properties (Cruz et al., 2006). Peptides designed based on this epitope can elicit a strong immune response in mice and the antibodies generated can effectively neutralize PEDV (Cruz et al., 2008). Two non-neutralizing epitopes, SS2 (748YSNIGVCK755) and SS6 (764LQDGQVKI771), were detected at specific sites in the S protein (Sun D. et al., 2008). Meanwhile, Li et al. (2022) constructed recombinant plasmids pET-28a-S, pET-28A-S-SEA, and pET-28A-S-EP-SEA and evaluated the immunogenicity of their recombinant proteins. The purified recombinant protein immunized mice produced strong humoral and cellular immunity, among which the level of recombinant protein S-EP-SEA was the highest.

### 6.2 Transgenic vaccine

The S gene is the principal immunogenic gene of PEDV. The encoded S protein harbors numerous conserved neutralizing antigen epitopes, making the S gene a pivotal contender for the advancement of genetically modified vaccines (Kirchdoerfer et al., 2021). The neutral epitope of the PEDV S protein is situated within the 499–638 aa. Additionally, the linear epitopes S1P1 (248–280 aa), S1P2 (442–499 aa), and S1P3 (697–742 aa) have been detected. Among these, the S1P3 epitope exhibits the highest antigenicity (Sun et al., 2008b). Furthermore, the N-terminal region of the S1 protein (18–233 aa) harbors crucial epitopes that elicit protective immune responses and facilitate binding to sialic acid receptors located on the cellular membrane (Hou et al., 2017).

In an attempt to examine the process of neutralizing the COE region of the S1 protein, Huy et al. (2012) fused the COE region of the PEDV S gene with the M cell-targeting ligand Co1. The vector containing this segment was introduced into rice calli to produce transgenic rice expressing the COE–Co1 fusion protein. Subsequent mouse-feeding studies showed that transgenic rice could trigger a strong systemic and mucosal immune response against the COE antigen (Huy et al., 2012). Meanwhile, Tien et al. (2021) linked the COE fragment of the PEDV S gene with the polymeric immunoglobulin scaffold (PIGS) to produce anti-COE IgG antibodies in the serum of mice orally immunized with the recombinant expression vector. Anti-COE IgA antibody was detected in the stool samples.

### 6.3 Liver carrier vaccine

Recombinant live vector vaccines are created by inserting the gene encoding the effective antigen protein into a live vector, causing it to express the protein (Han et al., 2021). The expression level and immunogenicity of vaccines prepared to target the S gene are not ideal. This may be related to the instability of the S gene structure. Therefore, establishing methods to stabilize the S gene structure would prove monumental for developing new vaccines (Bos et al., 2020).

Ge et al. (2012) established a connection between the PEDV S protein COE and *Escherichia coli* heat-labile enterotoxin B (LTB) to create a live carrier vaccine using *Lactobacillus casei*. After immunizing mice, the production of secretory immunoglobulin (Ig)A and IgG was stimulated, which could effectively neutralize the virus in the serum and mucus (Ge et al., 2012). Meanwhile, Ma et al. (2018) formulated a vaccine using oral recombinant *Lactobacillus casei* designed to target intestinal M cells and DCs and elicit mucosal immunity against PEDV. They reported the induction of humoral and cellular immune responses in mice. Chen et al. (2022) used the expression system of Drosophila embryo S2 cells to express the PEDV S1 protein. The purity of the antigens exceeded 80% and exhibited specific reactivity with PEDV-positive serum, which was present in the cell culture supernatant as a secreted protein, facilitating its separation and purification. The recombinant PEDV S1 protein was mixed with an adjuvant to immunize Kunming mice, inducing a highly effective PEDV-specific IgG response (Chen et al., 2022). In addition, Huang (2016) immunized mice with concatenated PEDV S-protein core epitopes expressed using the *E. coli* expression system and *E. coli* heat-labile enterotoxin. The recombinant S1 protein expressed in the S2 cells of fruit fly embryos effectively induces a humoral immune response along with the reported cellular immune responses (Chen et al., 2022).

Bae et al. (2003) developed transgenic tobacco plants expressing the neutralizing epitope of the PEDV S protein, demonstrating the notable contribution of the S protein to the viral immune response. Feeding these plants to mice generated systemic and mucosal immune responses. The generated antibodies could impede PEDV proliferation on cellular substrates (Bae et al., 2003). In another study, a live carrier vaccine expressing the PEDV S1 protein was constructed using *Lactococcus lactis* as the carrier, resulting in the production of effective neutralizing antibodies (Liu et al., 2012). Reports, on expressing the PEDV S1 region or regions containing neutral epitopes using various methods, all of which have shown good immunogenicity, are available (Oh et al., 2014). Zhang et al. (2021) successfully inserted PEDV S genes into non-coding regions downstream of three PRV open reading frames (UL11-10, UL35-36, and UL46-27), resulting in recombinant viruses that stably expressed the S protein. In addition, they found that immunization of weaned piglets with a recombinant virus vaccine generated via the insertion of the S gene into the non-coding region between UL40-41 of the PRV genome induced the production of low levels of PEDV antibodies (Zhang et al., 2023). Li et al. (2021) also developed a recombinant strain, L-casei-OMP16-PEDV S that expressed the PEDV S protein and Brucella DMP16 protein based on *Lactobacillus casei*. ELISA analysis showed that the expression of interleukin (IL)-4, IL-10, and interferon (IFN)-γ in immunized mice was enhanced and the level of IgA secretion was significantly increased, suggesting that the recombinant *Lactobacillus casei* had good immunogenicity. Additionally, Liu et al. (2019) constructed a novel recombinant adenovirus rAd-PEDV-S expressing PEDV S to immunize 4-week-old Suckling pigs, effectively inducing PEDV-specific humoral immune responses.

Intestinal bacteria have been used to develop live-carrier vaccines to induce specific cellular and humoral immunity in the intestinal immune cells. Lactic acid bacteria, *Bacillus subtilis*, and *Salmonella* spp. are commonly used as live bacterial carriers. Zang et al. (2020) transferred PEDV S protein S1 and S2 epitope recombinant plasmids into porcine *Lactobacillus acidophilus* to prepare an oral vaccine administered to BALB/c mice. They reported induction of PEDV-specific SIgA levels significantly higher than those in the commercial inactivated vaccine group. Wang et al. (2019) employed recombinant *B. subtilis* to produce the COE antigen of the PEDV S protein and demonstrated its potent immunogenicity through oral immunization of piglets. Meanwhile, Huy et al. (2016) used tobacco cells to express PEDV S1D epitopes (636–789 aa) and a cholera toxin B (CTB) subunit protein. After oral immunization of mice, IgG and IgA antibodies against S1D and CTB were induced. Wang et al. (2017) prepared a fusion protein containing a PEDV COE and a DC-targeting peptide using lactic acid bacteria. After oral immunization of mice, the fusion protein effectively induced mucosal and humoral immune responses. Additionally, Li et al. (2021) used recombinant Lactobacillus to express a fusion protein comprising the capsid protein of PCV2 and LTB, which produced significant mucosal and systemic immune responses to PCV2 in mice following oral immunization. Previous studies demonstrated the feasibility and safety of porcine pseudorabies virus (PRV) as a live carrier for PEDV (Xu et al., 2004, 2009). Proteins encoded by exogenous genes in recombinant PRV exhibit natural protein activities that can induce the production of humoral and cellular immune responses for an extended duration. Therefore, constructing a recombinant PRV that can efficiently express PEDV S1 protein is feasible (Hou et al., 2023).

Not all epitopes in the S protein can induce neutralizing antibodies, as the intact S or S1 protein contains many epitopes that induce non-neutralizing antibodies, which may include those associated with antibody-dependent enhancement (Yu et al., 2021; Thavorasak et al., 2022). Neutralizing antibodies that specifically target the RBD of the S protein play a crucial role in inhibiting the interactions between the S protein and its receptor. Lao et al. (2024) successfully prepared a recombinant porcine reproductive and respiratory syndrome virus gene-engineered live carrier vaccine candidate strain named rHuN4-F112-SNE. After purification and screening, this strain expressed the dominant antigen region, known as the SNE locus, of the PEDV S protein. They then verified that the introduction of the SNE gene does not affect the replication of the modified virus and that the SNE protein is expressed through recombinant virus infection. These effects are favorable for inducing specific antibodies, indicating the feasibility of using a live carrier vaccine against PEDV (Lao et al., 2024). Zou et al. (2023) further engineered a genetically modified Sendai virus capable of expressing PEDV S protein. The recombinant virus induced PEDV-specific humoral immunity, cellular immunity, and neutralizing antibody responses in mice.

Adenoviruses are suitable vectors for expressing foreign genes due to their wide host range and ability to efficiently express multiple genes in a short time. Do et al. (2020) constructed a recombinant adenovirus vaccine rAd-LTB-COE encoding heat-labile enterotoxin B (LTB) and PEDV COE. Intramuscular or oral administration of the vaccine induced strong humoral and mucosal immune responses in piglets, with the level of S-protein-specific IgG significantly higher in immunized piglets than in the PBS control group.

### 6.4 Subunit vaccine

The first step in the development of PEDV subunit vaccines is the selection of suitable antigen targets. S protein represents the primary antigenic protein of PEDV and, thus, the primary focus of PED subunit vaccines (Liu et al., 2024). In addition, designing the S protein structure is a technical route for the development of the novel coronavirus subunit vaccine; its recombinant S protein expression product exhibits good antigenicity, providing a reference for the development of PED subunit vaccines (Hsieh et al., 2020).

The S protein contains multiple B-lymphocyte antigen epitopes that induce a strong humoral immune response. Therefore, the development of novel PEDV vaccines has focused primarily on the PEDV S protein. Currently, inactivated or live attenuated vaccines for PEDV are extensively utilized for immunization in clinical practice and have shown good prevention and control effects (Yin et al., 2021). However, with the continuous mutation of the virus and the emergence of the mutant G2b subtype strain, pressure to achieve effective prevention and control of PEDV has increased (Guo et al., 2016; Tang et al., 2020). Meanwhile, attenuated vaccines are also limited by detoxification or virulence, which can increase biosafety risks. Wang et al. (2024) successfully constructed CHO cell lines that stably and exogenously express PEDV S. Approximately 5 mg of PEDV S protein can be obtained from 100 mL of CHO-PEDV/S cell culture supernatant. This has improved the expression and secretion of PEDV S protein, laying a foundation for further development of PEDV subunit vaccines.

Contrary to attenuated vaccines, subunit vaccines have numerous benefits, including strong antigen specificity, good immunogenicity, and high biosafety (Wang et al., 2018; Zhao et al., 2018). Hsu et al. (2020) constructed VLPs expressing PEDV-containing S protein, M protein, and E protein; the system became effectively induced in immunized weaned piglets to produce anti-PEDV-specific IgG and cellular immune responses. Meanwhile, Tan et al. (2014) inserted the PEDV S1 gene into a *Pichia pastoris* expression vector and confirmed that the expressed target protein was specifically bound to PEDV-positive serum. Masuda et al. (2021) successfully produced a trimeric PEDV S protein using the *Bombyx baculovirus* expression vector system. Subsequent administration to mice resulted in detectable levels of anti-S protein-specific IgG in the bloodstream, capable of neutralizing the virus and protecting Vero cells from PEDV infection (Masuda et al., 2021). Sun et al. (2021) produced a PEDV S-B recombinant protein using *Pseudomonas aeruginosa* exotoxin A lacking domain III as a carrier; upon intraperitoneal administration to mice, a targeted humoral and T helper 1 cell-type predominant cellular immune response was elicited. Still further, Li et al. (2022) combined recombinant Salmonella flagellin (rSF) with a COE located on the PEDV S1 subunit to create an rSF–COE–D fusion protein. The diarrhea rate in piglets that were administered this fusion protein was significantly reduced after infection challenge. Xiu (2023) optimized a PEDV subunit vaccine and found that, compared with the mice with truncated proteins, PEDV-S trimer immunized mice induced stronger neutralization. Moreover, the serum immunized with S protein of the current G2 subtype strain exhibited neutralizing protection against the G1 subtype strain. Fu et al. (2024) used a HEK-293F cell eukaryotic expression system to express different truncated proteins of the PEDV S gene and further evaluated the titer of antibodies generated against the PEDV subunit vaccine. The results showed that the PEDV S protein had the best immunogenicity.

### 6.5 Nucleic acid vaccine

Subunit vaccines prepared with the PEDV S protein are considered safe; however, they often achieve weak immunogenicity and a low effective protection rate in immune animals. Therefore, such vaccines must be developed in combination with effective adjuvants to enhance their immunogenicity (Li et al., 2022). Meanwhile, PEDV S nucleic acid vaccines are created by directly introducing a plasmid to carry the S gene into animal somatic cells. The host cell expression system is then utilized to synthesize the antigen protein, which in turn induces the host to produce an immune response against the anti-S protein (Ma, 2023). Meng et al. (2013) used the eukaryotic expression vector pIRES to produce open reading frames that encode the S1 protein of TGEV; intradermal inoculation of mice resulted in an increase in the population of CD4+ and CD8+ T lymphocyte subsets and elicited elevated levels of IFN-γ. Meanwhile, immunization of mice with the DNA vaccine produced by Yin et al. (2019) (i.e., pPI-2.EGFP.VP7) via intramuscular or subcutaneous injection produced specific antibodies, increased IFN-γ and IL-4 secretion, and heightened the proliferation of splenic lymphocytes. Similarly, Suo et al. (2012) constructed a PEDV-S DNA vaccine and examined its effect on T cell expansion, anti-PEDV antibody production, IFN-γ and IL-4 expression, and cytotoxic T cell activity in the peripheral blood and spleen of Kunming mice, confirming the elicitation of a strong immune response.

Compared with subunit vaccines, nucleic acid vaccines have a simpler preparation process and elicit a more comprehensive immune response. However, the immune efficacy of nucleic acid vaccines is significantly influenced by the vaccination method. The PEDV S recombinant live carrier vaccine reassembles the S gene, which encodes an effective antigen protein, into a live virus or live bacterial vector, facilitating its expression. Hain et al. (2016) developed a recombinant paracoxvirus, ORFV PEDV-S capable of expressing the PEDV S protein. Intramuscular injections were administered to piglets to induce anti-PEDV serum IgG, IgA, and neutralizing antibody responses (Hain et al., 2016). Following the administration of inoculations to gestating sows, piglets born 3 days later had detectable levels of PEV-specific IgG, IgA, and neutralizing antibodies. In addition, morbidity and mortality significantly decreased after the PEDV outbreak (Joshi et al., 2018).

### 6.6 Inactivated vaccine

Recent research on PED vaccines has primarily focused on inactivated vaccines. Live vaccines are mainly administered orally, whereas inactivated vaccines are predominantly administered via intramuscular injection (Li, 2023). The primary antigen of the inactivated PED vaccine is considered to be the S1 protein, which is linked to the neutralizing epitope of S1. The vaccine based on the full-length S1 protein can provide 87.5–100% protection, whereas the immune protection of a vaccine based on the S1-D region is only 50%. In addition, studies have found that the SINDEL strain (Opriessnig et al., 2017) and nsp162′-O-M methyltransferase active site mutant strain (Hou et al., 2019) can serve as candidates for attenuated vaccines. Inoculation with these strains can increase the piglet survival rate to 100%. When altering the virulence genes of the isolated PEDV strains using reverse genetics, the resulting attenuated virus did not show differences in protein structure or immunogenicity compared to its parent strain S1 (Li, 2023).

Gillam and Zhang (2018) used the inherent self-assembly properties of the hepatitis B virus core protein (HBc) to prepare four virus-like particle vaccines. The HBc antigen vaccine neutralized the PEDV CO strain; however, after intranasal immunization of mice, antibody production in response to the four vaccines was lower than that of the control HBc group. Abdominal immunization with vaccines containing the 748–755 aa and tandem epitopes produced antibodies with a certain degree of neutralizing effects (Gillam and Zhang, 2018).

The S protein of PEDV is cleaved to form the S1 and S2 domains essential for the identification of host cell receptors and membrane fusion. The S protein is commonly used as an antigen for the development of coronavirus vaccines due to the presence of the S1 subunit (Nguyen Thi et al., 2022; Thavorasak et al., 2022). The PEDV S1 protein can be produced using the HEK-293F cell eukaryotic expression system and Drosophila embryonic S2 cells. After purification, subcutaneous injection of the protein into mice induced highly potent neutralizing antibodies and promoted cellular immune responses (Chen et al., 2022; Fu et al., 2024). Wu (2022) utilized lentivirus and recombinant adenovirus expression systems to express the S1 protein and immunize Bama miniature pigs, demonstrating that both vaccines could elicit specific mucosal immune responses. Egelkrout et al. (2020) constructed a corn structure of the epPEDV S1 protein and found that it only induced the production of serum-neutralizing antibodies after oral immunization in pigs. Xiao (2022) engineered *Lactobacillus casei* with a genome expressing the PEDV S1 gene; oral immunization of mice and piglets induced mucosal, humoral, and cellular immune responses, along with the production of neutralizing antibodies. Guo et al. (2019) constructed a *Lactococcus lactis* strain expressing PEDV S1. Following immunization of mice, the levels of PEDV-specific serum IgG and mucosal secreted IgA (sIgA) antibodies significantly increased. Moreover, Nie et al. (2021) engineered a *Lactobacillus* strain expressing PEDV S1; oral immunization of guinea pigs with sIgA antibodies and specific neutralizing antibodies stimulated the production of IL-4 and IFN-γ. Ma (2023) constructed four recombinant *Lactobacillus* strains as host bacteria expressing the PEDV S1 protective antigen, which induced specific mucosal, humoral, and cellular immune responses in juvenile pigs. This construct also effectively colonized the jejunum of newborn piglets and stimulated the intestinal immune system of pigs to produce PEDV mucosal antibodies for an extended period (Ma, 2023).

Although the current vaccines provide a certain level of protection, the vaccine strain is not effective against PEDV due to the substantial genetic diversity between the vaccine strain and currently prevalent wild strains, especially with respect to mutations or deletions in the S gene. Consequently, the current primary research focus is to create safer and more potent vaccines that utilize currently prevalent strains to effectively prevent PEDV (Wen, 2020). SIgA can improve the ability of the piglets to neutralize PEDV infection, reduce mortality, and minimize losses (Langel et al., 2020). IgG and sIgA in breast milk are the only forms of immunity acquired in piglets. The production of sIgA is essential for stimulating mucosal immunity in the sow’s intestine and activating the so-called “gut–mammary gland–sIgA axis” (Ohno, 2016). Ensuring that the development of an effective PED vaccine maintains the immunogenicity of the viral S protein enhances the mucosal immune protection of the host organism, induces the body to produce sufficient sIgA, and provides adequate antibody protection for piglets through the colostrum and breast milk, is crucial.

The current basic strategy for controlling and eradicating PED outbreaks involves the vaccination of pregnant sows (Langel et al., 2016). A wide range of commercial vaccines are extensively employed to prevent and manage PED in China, South Korea, and Japan. Following the onset of PED in North America in 2013, multiple additional vaccines were introduced (Wen, 2020).

Several studies have also demonstrated the importance of ORF3 in determining the virulence of PEDV. However, the specific mechanism through which ORF3 modulates the pathogenicity of the virus remains largely unelucidated (Park et al., 2008). ORF3 is predicted to have a structural configuration comprising multiple transmembrane domains that serve as tetrameric ion channels (Wang et al., 2012). Given that it is difficult to obtain the complete ORF3 protein from isolated PEDV, performing a detailed study of its function has proven challenging. Assessing the expression of ORF3 in virus-infected cells presents a major challenge in current research (Kaewborisuth et al., 2018).

To overcome these challenges, Kaewborisuth et al. (2018) created a recombinant PEDV strain containing ORF3 tagged with Myc to examine the influence of ORF3 on PEDV. Using flow cytometry and confocal microscopy, they discovered that an ORF3 protein with an N-terminal label was also present on the cell surface of the PEDV S protein. Therefore, the authors speculated an interaction between the helper protein ORF3 and the S protein. To confirm this hypothesis, they transfected pCAGGS-ORF3-FL or Trnc and pCAGGS-SAV12 vector with recombinant PEDV and analyzed the transfected cells using an immunofluorescence assay. Co-immunoprecipitation was performed in cells simultaneously expressing ORF3 and S proteins. The results showed that both ORF3 variants precipitated together with the S proteins. Furthermore, two proteins showed higher concentrations in the perinuclear membranes and vesicle-like compartments of transfected cells. These experiments thus confirmed the interaction between ORF3 and S proteins (Kaewborisuth et al., 2018). However, the ultrastructural characteristics of PEDV-infected cells indicate that the replication sites of PEDV are mainly located in the perinuclear space. Mature viral particles can be formed, transported, and packaged into vacuoles through vesicular pathways in the endoplasmic reticulum and Golgi compartment (Zhou et al., 2017). ORF3 and S proteins were present simultaneously in the perinuclear region and vesicle structures, indicating that the interaction between these proteins primarily influences PEDV replication. However, the specific interaction mechanism between S and ORF3 proteins was not clarified (Kaewborisuth et al., 2018). At this stage, only the interaction between the ORF3 and S proteins has been confirmed and relatively few studies have investigated interactions with other viral proteins. Therefore, further studies should investigate the detailed interaction and mechanism between the PEDV S protein and other viral proteins.

## 7 Application of S protein in PEDV diagnosis

The current diagnostic technology for PEDV has advanced. The diagnosis of porcine epidemic diarrhea can usually be made with laboratory and clinical diagnoses. Porcine epidemic diarrhea may be suspected when the symptoms are more typical. For example, after infection, the early feces of Suckling piglets are yellowish-brown and sticky and watery in the middle and late stages (Hou and Shan, 2023). Necropsy of suspected diseased pigs showed dilated intestines to varying degrees, translucent, with a large amount of liquid contents, intestinal lymph nodes showed obvious swelling and bleeding, and intestinal chorionic membranes showed atrophy and detachment (Gao, 2022). These symptoms can be a preliminary diagnosis of PED; however, the specific diagnosis requires laboratory diagnostic methods.

There are three types of diagnosis: pathogenic, immunological, and molecular biology. Isolating and identifying viruses is an important means of diagnosing viral diseases in livestock and poultry. The results are direct, reliable, and accurate (Gao, 2022). Common laboratory diagnostic methods include neutralization test (NA), enzyme-linked immunosorptive assay (ELISA), RT-PCR, electron microscopy and immunoelectron microscopy, colloidal gold immunochromatography, and mixed detection methods (Luan, 2024).

The S protein on the outer surface of PEDV plays a key role in PEDV recognition and fusion of other proteins; hence, it is often a key in the diagnosis of PEDV. The PEDV S protein contains numerous epitopes capable of eliciting the production of neutralizing antibodies within the body, such as 499–638 aa, 748–755 aa, 764–771 aa, and 1,368–1,374 aa (Cruz et al., 2006). Based on this, substantial research has been conducted on PEDV-S monoclonal antibodies and epitopes, yielding over 70 variations in monoclonal antibodies that specifically target S proteins. These monoclonal antibodies can be utilized in various assays such as indirect immunofluorescence, protein immunoblotting (i.e., western blot), and enzyme-linked immunosorbent assay (ELISA). Among the more than 70 S protein monoclonal antibodies developed to date, only PC10 is a porcine monoclonal antibody, while the remaining are murine monoclonal antibodies (Zhang and Peng, 2022). The preparation of monoclonal antibodies can help to facilitate the serological detection of PEDV in diagnosis. Given the critical nature of PED prevention and control, it is imperative to produce PEDV antigen proteins with high purity and antigenicity (Li et al., 2023). Fu et al. (2017) selected single B lymphocytes secreting anti-PEDV S protein-specific antibodies from mesenteric lymph nodes following immunization with a PEDV vaccine. The single-cell polymerase chain reaction was used to amplify IgG heavy and light chain genes in isolated B cells, which were then expressed in 293 T cells to produce PC10, in turn, PC10 can specifically bind to G1 and G2 PEDV and effectively neutralize PEDV infections (Fu et al., 2017). Liu et al. prepared several strains of monoclonal antibodies, among which 1B9 (Liu et al., 2017), 2B11, 1E3 (Gong et al., 2018), 8A3A10 (Zhang et al., 2016), and 2E10 (Kong et al., 2020) showed virus-neutralizing activity. Other researchers have screened multiple strains of monoclonal antibodies, including 8C3 (Leng, 2014), 1F2 (Wang C. F., 2014), and 4C7 (Gui et al., 2018), that can react with prokaryotic S proteins. Immunofluorescence assays indicated that the monoclonal antibodies 1D7, 2E11, 3G9, 4G5, 4A12, and 5G8 (Min, 2017), as well as E3, G8, and G9 (Wang et al., 2015), produced specific immunofluorescence in PEDV-infected Vero cells. Li et al. (2023) immunized BALB/c mice with PEDV S1 recombinant protein expressed in mammalian 293 T cells and obtained five monoclonal antibody-expressing cell lines that could react specifically with the PEDV S1 protein, namely 12D14H4, 13F5B9, 10D2B10, 11A7C9, and 14E6F5. Sun D. et al. (2008) and Sun et al. (2008a) identified the core sequences of two epitopes based on the prepared monoclonal antibody, while Zhu et al. (2013) amplified the variable region genes of monoclonal antibodies and successfully expressed a single-chain variable fragment antibody of the S protein with a GST tag.

To clarify the role of various regions of the S protein in PED diagnosis, Li et al. (2017) successfully located each of 10 monoclonal antibodies in a specific small region of the S1 domain. Four monoclonal antibodies targeted S10 (19–220 aa), two targeted S1A (220–510 aa) and S1B (510–640 aa), and one targeted S1CD (640–729 aa). The authors found that only the S10 and S1B regions could produce neutralizing antibodies in strongly mutated strains of PEDV and suggested that S10 acts as an effective epitope within the mutated strains, whereas an antigen epitope may be present within S1B that is effective against all PEDV genotypes. Therefore, the S10 and S1B epitopes from these mutated strains are potential vaccine candidates (Li et al., 2017).

Bai et al. (2022) successfully prepared rabbit polyclonal antibodies with good immunogenicity by expressing and purifying the PEDV S protein. Li (2022) successfully constructed prokaryotic expression recombinant plasmids pET-30a(+)-PEDV-S, pNus32T-PEDV-S1, and eukaryotic expression recombinant plasmids pFLAG-PEDV-S1a and successfully prepared polyclonal antibodies with a titer greater than 1:380,500 that could detect 5 ng of the target protein. The polyclonal antibody could recognize the PEDV-S1a protein expressed in eukaryotes and the PEDV-S1 protein expressed in prokaryotes.

Heavy-chain antibodies consist of only heavy chains (Hamers-Casterman et al., 1993). Variable domains of heavy-chain antibodies within the Camellidae family are referred to as VHH, which have a relative molecular mass of approximately 15 ku, diameter of approximately 2.5 nm, and height of approximately 4 nm. VHH is the smallest antibody possessing a fully functional antigen-binding site and is, therefore, referred to as a nanobody (Muyldermans et al., 2009). Compared with conventional antibodies, nanobodies have several advantages, including high stability, good solubility, strong affinity, low immunogenicity, easy expression and purification in *E. coli*, and a high expression yield (Yang et al., 2018; Salvador et al., 2019). The use of nanobodies targeting the PEDV S protein for the treatment and diagnosis of PED has been investigated. Wang et al. (2021) expressed and purified the PEDV S1 protein and subsequently constructed a diverse phage-display library by immunizing Bactrian camels. Moreover, phage-display technology was used to screen six nanobodies that specifically bind to the PEDV S1 protein. The authors provided valuable resources for detecting PEDV and developing nanobody-based drugs (Wang et al., 2021).

Etiological detection methods can quickly and accurately distinguish PEDV from other porcine intestinal pathogens. Serological detection techniques enable the assessment of the prevalence of PEDV infection, as well as immunization effectiveness. Among all serological detection methods, ELISA remains commonly used owing to its affordability, rapidity, safety, specificity, and sensitivity. However, these detection methods require further enhancements to be effectively implemented in practical applications. Progress in science and technology, along with extensive research on PEDV, will improve the existing detection technology or facilitate the development of a novel rapid detection technology for PEDV (Luan, 2020).

## 8 Future outlook

The S protein of PEDV, a transmembrane glycoprotein located on the surface of viral particles, primarily identifies and attaches to receptors on host cells, facilitating membrane fusion between the virus and the host. The S protein is comprised of neutralizing epitopes, signal peptides, transmembrane domains, and short cytoplasmic domains and can be cleaved into S1 and S2 subunits. The structural features of the S protein make it a crucial recognition protein located on the surface of PEDV. Similar to S proteins of other coronaviruses, the PEDV S protein is a glycoprotein peptide located on the surface of the virus and contains neutralizing epitopes that can induce the production of neutralizing antibodies, making it an important immunogenic protein. Moreover, the S protein is involved in the evasion of the immune system and may affect the immune responses of hosts. The S protein also affects PEDV virulence and can trigger cell apoptosis, although the precise mechanism by which it influences PEDV virulence remains nebulous, which necessitates further investigations.

PEDV is an RNA virus, hence has a very high mutation rate. With the spread and development of PEDV, viral particles have undergone several mutations. Consequently, the strains responsible for current PED outbreaks are mostly mutated strains with different gene sequences than those of the original strain. Therefore, the prevention and control of PEDV is important. In this review, we have highlighted the following five areas of focus for future research on the PEDV S protein.

(1) The S protein as a vaccine candidate. The S protein is an ideal candidate for the development of a vaccine against PEDV due to its crucial role in host cell recognition and invasion related to its immune properties and ability to evade the immune system. Several vaccine formulations, including PEDV recombinant protein vaccines, adenovirus vector vaccines, and other genetically engineered vaccines, have been developed using the S protein. Furthermore, inactivated vaccines containing S proteins have been developed. Future studies should evaluate the safety and effectiveness of S protein-based vaccines as well as broaden the spectrum of S protein-associated vaccines to enhance the prevention and control of PEDV infection and transmission.
(2) Expanding the research on the structure and function of the S protein to better understand its interaction with host cells and involvement in the genetic variation and evolution of the virion. These investigations will establish a crucial theoretical basis for the future development of novel anti-PEDV medications and vaccines.
(3) Investigating the interactions between the S protein and other viral proteins of PEDV to gain deeper insights. Currently, data regarding the influence of S proteins on host proteins and their receptors, as well as their interactions with other viral proteins, are lacking. Therefore, further investigation of the mechanisms underlying these interactions is crucial for a more thorough understanding of their function and role within PEDV virions. This will provide valuable insights into the viral life cycle and transmission mechanisms, ultimately contributing to the development of effective vaccines against PEDV.
(4) Investigating the role of the S protein in PEDV diversification and evolution. The currently circulating strains of PEDV have acquired several mutations compared with the original strain. Accordingly, the impact of vaccines developed based on previous strains is significantly reduced in combatting the current strains. Therefore, it is imperative to understand the diversity and progression of PEDV to develop potent vaccines and best manage the virus. Subsequent research is therefore required on the precise function and mechanism of the S protein in the mutation and evolution of viruses. This will facilitate the discovery of newly emerged mutant strains and implement effective prevention and control measures. These developments will significantly influence the prevention and management of PEDV infections.
(5) Investigating the effects of the S protein on PEDV replication. Contemporary research on PEDV structural proteins has revealed the involvement of the N, M, E, and ORF3 helper proteins in viral replication. Furthermore, the interaction between the S protein and other viral proteins substantiates the potential collaboration between ORF3 and S proteins to facilitate viral replication. Consequently, future investigations should focus on elucidating the role of S protein in promoting viral replication. Understanding the function and specific mechanism of action of the S protein will help to advance vaccine development.

As the most important structural protein on the surface of PEDV virions, the S protein affects PEDV virions by influencing pathogenicity, the body’s immune response, and inducing cell apoptosis, among other effects. However, the function and mechanism of action of PEDV remain unclear. The S protein located on the surface of PEDV virions is a crucial determinant of viral behavior; therefore, understanding the interactions of the S protein with receptors and proteins is crucial for the development of a PEDV vaccine. Moreover, gaining a thorough understanding of the specific mechanisms of action of the S protein is crucial for the prevention and treatment of PEDV infection. Further research in this field will have profound importance for the global pig industry.

## Author contributions

HL: Writing – original draft. ZL: Writing – original draft. JL: Writing – original draft. YW: Writing – original draft. MZ: Writing – review & editing. SH: Writing – review & editing. YL: Writing – original draft. KM: Writing – original draft.
